# Complete Coding Genome Sequence of an Avian Influenza A/H3N8 Virus Strain Detected in North Kazakhstan in 2018

**DOI:** 10.1128/MRA.00441-20

**Published:** 2020-07-16

**Authors:** Kulyaisan Sultankulova, Mukhit Orynbayev, Nurlan Kozhabergenov, Karlygash Akylbayeva, Aibarys Melisbek, Kuanysh Jekebekov, Asankadyr Zhunushov, Kunsulu Zakarya, Yerbol Burashev

**Affiliations:** a Research Institute for Biological Safety Problems (RIBSP), Gvardeyskiy, Kazakhstan; b Institute of Biotechnology of the National Academy of Sciences of the Kyrgyz Republic, Bishkek, Kyrgyzstan; KU Leuven

## Abstract

We report the complete coding genome sequence of the influenza A/H3N8 virus, isolated from Anas querquedula in northern Kazakhstan in 2018. Phylogenetic analysis of the surface antigens of strain A/garganey/North-Kazakhstan/45/2018 showed that its hemagglutinin belonged to the Asian line, while its neuraminidase was assigned to the Eurasian group.

## ANNOUNCEMENT

Influenza A virus belongs to the genus *Alphainfluenzavirus* of the virus family *Orthomyxoviridae*. Subtyping of influenza A virus occurs according to the antigenic specificity of surface glycoproteins. For birds, the most pathogenic variant is the type A/H5N1 (1). Based on the hemagglutinin (HA) and neuraminidase (NA), 18 H and 11 N subtypes, respectively, are currently known (2). Since 2005, a panepizootic epidemic of avian influenza caused by the highly pathogenic H5N1 strains was reported in many countries, most likely spread by wild migratory and waterfowl birds (3). According to the World Organization for Animal Health (OIE), it was noted that since 2013, a second panepizootic wave of bird flu has been observed (https://www.who.int/influenza/human_animal_interface/influenza_h7n9/140225_H7N9RA_for_web_20140306FM.pdf?ua=1). The situation is complicated by the circulation of various subtypes of the virus, which complicates the control and elimination of outbreaks. In January 2018, 8 countries (Afghanistan, Cambodia, Taiwan, Iraq, Japan, South Korea, Saudi Arabia, and South Africa) and two continents (Africa and Asia) were affected by outbreaks among poultry (4).

In order to determine the circulation of the avian influenza virus in the territory of the Republic of Kazakhstan, monitoring was carried out, as a result of which, in the autumn of 2018, 90 samples of cloacal swabs from wild birds from the North Kazakhstan region were delivered for laboratory studies. The viral RNA was extracted from cloacal swabs using the QIAmp viral RNA minikit (Qiagen, Germany) according to the manufacturer’s instructions. Real-time PCR (RT-PCR) was used for primary detection of type A influenza virus in cloacal swabs. RT-PCR targeted the highly conserved region of the M gene, according to the method of Spackman et al. (5), using the RT-PCR system LightCycler version 2.0 (Roche Applied Science, Germany).

As a result, sample number 45 showed a positive result around cycle 30 and was used for subsequent studies. All eight gene segments were amplified in the SuperScript one-step RT-PCR system with Platinum *Taq* DNA polymerase (Invitrogen SRL) using Uni-12 (3′-UCG YUU UCG UCC) and Uni-13 (GG AAC AAA GAU GA-5′) universal influenza primers according to the Hoffmann method (6). Sequencing was carried out in a 16-capillary genetic analyzer AB3130xl (Hitachi Applied Biosystems) with a BigDye Terminator cycle sequencing kit version 3.1 (ABI, Foster City, CA, USA). Raw data were processed with the use of Sequencher version 5 (GeneCodes Corp.) and BioEdit version 7.2.5 for sequence assembling and alignment. The size of each virus segment is shown in Table 1.

**TABLE 1:** Genome characteristics of strain A/garganey/North-Kazakhstan/45/2018

Gene/segment	Size (nucleotides)	GC content (%)	Strain with closest relative sequence	Identity at nucleotide level (%)	GenBank accession no.
PB2	2,338	44.8	A/barnacle goose/Netherlands/2/2014	99.0	MT126625
PB1	2,323	44.4	A/mallard duck/Georgia/10/2016	98.8	MT126633
PA	2,200	44.1	A/black-tailed godwit/ Bangladesh/24734/2015	98.8	MT126634
HA	1,763	43.6	A/greylag goose/North-Kazakhstan/62/2019	99.9	MN945300
NP	1,551	47.3	A/teal/Egypt/MB-D-487OP/2016	99.2	MT126635
NA	1,264	45.8	A/northern shoveler/North-Kazakhstan/20/2018	99.2	MN945299
M	1,027	49.3	A/northern shoveler/Egypt/MB-D-695C/2016	99.5	MT126636
NS	890	42.9	A/greater white-fronted goose/Netherlands/11/2009	99.1	MT126637

**FIG 1:**
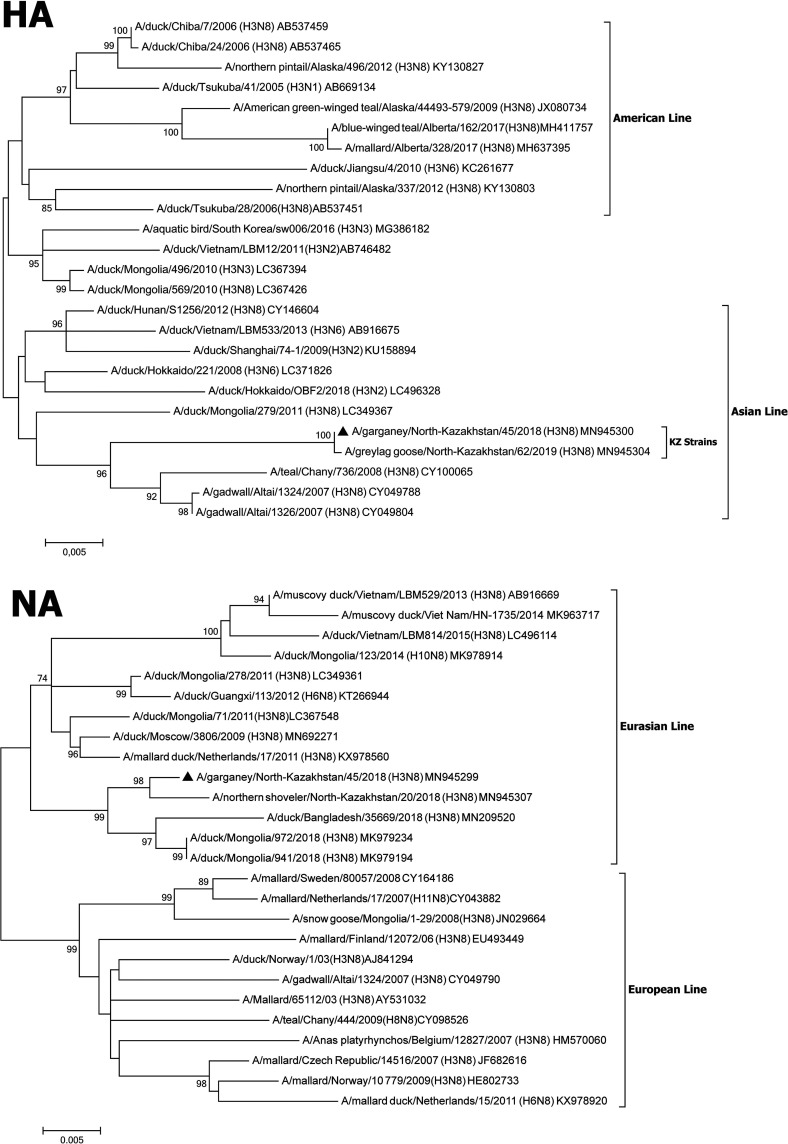
Phylogenetic tree for HA and NA genes of the strain A/garganey/North-Kazakhstan/45/2018 (H3N8). Phylogeny of the HA and NA genes was inferred using the maximum likelihood method with 1,000 bootstrap replicates in MEGA7. At each branch, the number indicates a bootstrap value (>70%). The Kimura 2-parameter substitution model was selected with the assumption of a gamma distribution with invariant rates among sites (7). The location of the sequence reported here is indicated with a black triangle.

The H3N8 subtype was determined based on the results of a comparative analysis of surface antigens using BLAST. Phylogenetic analysis of surface antigens of strain A/garganey/North-Kazakhstan/45/2018 showed that HA belongs to the Asian line, while NA was assigned to the Eurasian group (Fig. 1).

### Data availability.

The complete coding genome sequence of strain A/garganey/North-Kazakhstan/45/2018 (A/H3N8) was published in GenBank under the following accession numbers: MN945300, MN945299, MT126625, and MT126633 to MT126637.

## References

[1] Prosser DJ, Cui P, Takekawa JY, Tang M, Hou Y, Collins BM, Yan B, Hill NJ, Li T, Li Y, Lei F, Guo S, Xing Z, He Y, Zhou Y, Douglas DC, Perry WM, Newman SH 2011 Wild bird migration across the Qinghai-Tibetan plateau: a transmission route for highly pathogenic H5N1. PLoS One 6:e17622. doi:10.1371/journal.pone.0017622. 21408010 PMC3052365

[2] Fouchier RAM, Munster V, Wallensten A, Bestebroer TM, Herfst S, Smith D, Rimmelzwaan GF, Olsen B, Osterhaus ADME 2005 Characterization of a novel influenza A virus hemagglutinin subtype (H16) obtained from black-headed gulls. J Virol 79:2814–2822. doi:10.1128/JVI.79.5.2814-2822.2005. 15709000 PMC548452

[3] Burashev Y, Strochkov V, Sultankulova K, Orynbayev M, Kassenov M, Kozhabergenov N, Shorayeva K, Sadikaliyeva S, Issabek A, Almezhanova M, Nakhanov A, Savitskaya I, Zakarya K 2020 Near-complete genome sequence of an H5N1 avian influenza virus strain isolated from a swan in Southwest Kazakhstan in 2006. Microbiol Resour Announc 9:e00016-20. doi:10.1128/MRA.00016-20. 32217669 PMC7098892

[4] Williams RA, Peterson AT 2009 Ecology and geography of avian influenza (HPAI H5N1) transmission in the Middle East and northeastern Africa. Int J Health Geogr 8:47. doi:10.1186/1476-072X-8-47. 19619336 PMC2720944

[5] Spackman E, Senne DA, Myers TJ, Bulaga LL, Garber LP, Perdue ML, Lohman K, Daum LT, Suarez DL 2002 Development of a real-time reverse transcriptase PCR assay for type A influenza virus and the avian H5 and H7 hemagglutinin subtypes. J Clin Microbiol 40:3256–3260. doi:10.1128/JCM.40.9.3256-3260.2002. 12202562 PMC130722

[6] Hoffmann E, Stech J, Guan Y, Webster RG, Perez DR 2001 Universal primer set for the full-length amplification of all influenza A viruses. Arch Virol 146:2275–2289. doi:10.1007/s007050170002. 11811679

[7] Kumar S, Stecher G, Tamura K 2016 MEGA7: Molecular Evolutionary Genetics Analysis version 7.0 for bigger datasets. Mol Biol Evol 33:1870–1874. doi:10.1093/molbev/msw054. 27004904 PMC8210823

